# A Novel Moderate Constitutive Promoter Derived from Poplar (*Populus tomentosa* Carrière)

**DOI:** 10.3390/ijms14036187

**Published:** 2013-03-18

**Authors:** Zhong Chen, Jia Wang, Mei-Xia Ye, Hao Li, Le-Xiang Ji, Ying Li, Dong-Qing Cui, Jun-Mei Liu, Xin-Min An

**Affiliations:** National Engineering Laboratory for Tree Breeding (NDRC), Key Laboratory of Genetics and Breeding in Forest Trees and Ornamental Plants (MOE), the Tree and Ornamental Plant Breeding and Biotechnology Laboratory (SFA), College of Biological Science and Biotechnology, Beijing Forestry University, Qinghua Eastern Road No.35, Haidian District, Beijing 100083, China; E-Mails: sharazhonger@gmail.com (Z.C.); jiajia87519@163.com (J.W.); yemeixia123@163.com (M.-X.Y.); fire0329@163.com (H.L.); thomasji26@hotmail.com (L.-X.J.); liying105527@126.com (Y.L.); cuidongqing1986@126.com (D.-Q.C.); liujunmei2008@gmail.com (J.-M.L.)

**Keywords:** constitutive promoter, moderate expression, RT-PCR, GUS, transgenic plants

## Abstract

A novel sequence that functions as a promoter element for moderate constitutive expression of transgenes, designated as the *PtMCP* promoter, was isolated from the woody perennial *Populus tomentosa*. The *PtMCP* promoter was fused to the *GUS* reporter gene to characterize its expression pattern in different species. In stable *Arabidopsis* transformants, transcripts of the *GUS* reporter gene could be detected by RT-PCR in the root, stem, leaf, flower and silique. Further histochemical and fluorometric GUS activity assays demonstrated that the promoter could direct transgene expression in all tissues and organs, including roots, stems, rosette leaves, cauline leaves and flowers of seedlings and maturing plants. Its constitutive expression pattern was similar to that of the CaMV35S promoter, but the level of GUS activity was significantly lower than in CaMV35S promoter::*GUS* plants. We also characterized the promoter through transient expression in transgenic tobacco and observed similar expression patterns. Histochemical GUS staining and quantitative analysis detected GUS activity in all tissues and organs of tobacco, including roots, stems, leaves, flower buds and flowers, but GUS activity in *PtMCP* promoter::*GUS* plants was significantly lower than in CaMV35S promoter::*GUS* plants. Our results suggested that the *PtMCP* promoter from poplar is a constitutive promoter with moderate activity and that its function is presumably conserved in different species. Therefore, the *PtMCP* promoter may provide a practical choice to direct moderate level constitutive expression of transgenes and could be a valuable new tool in plant genetic engineering.

## 1. Introduction

Constitutive promoters direct expression in most or all tissues irrespective of environmental or developmental factors. As their expression is normally not affected by endogenous factors, constitutive promoters are usually active across species or even kingdoms. The most widely used promoter for directing constitutive expression in transgenic plants is the CaMV35S promoter, which was isolated from the cauliflower mosaic virus [1]. The CaMV35S promoter and its derivatives can drive high levels of foreign gene expression in dicots [2–6]. Many promoters from plants are also commonly used for their ability to drive the constitutively high expression of foreign genes, including *ZmUbi1*[7–9] from maize (*Zea mays*), *Gmubi*[10,11] from soybean (*Glycine max*), *ubi4* and *ubi9*[12,13] from Sugarcane (*Saccharum officinarum*), *ubi7*[14,15] from potato (*Solanum tuberosum*), *tCUP*[16–18] from tobacco (*Nicotiana tabacum*) and *Act1*[19,20], *GOS2*[21], *OsTubA1*[22], *OsCc1*[23], *RUBQ1* and *2*[24], *rubi3*[9] and *OsAct2*[25] from rice (*Oryza sativa*).

Many constitutive promoters are used to drive ectopic gene expression *in vivo* and *in vitro* contexts [26]. Constitutive promoters are frequently introduced into basic vectors for plant transformation to promote the expression of transgenes throughout the whole plant for many purposes, such as enhancing disease resistance [27,28], abiotic stresses tolerance [29,30] and herbicide and antibiotic resistance [31] and also play an important role in vaccines [32]. To achieve these certain traits, it is essential to use a strong constitutive promoter that can drive high level expression of foreign genes in most tissues.

Among constitutive promoters, the CaMV35S promoter is a prominent example and is most widely used in transgenic plants. Although it has many advantages and is a convenient tool for genetic engineering research, there are some potential weaknesses in its application. With the CaMV35S promoter, the foreign gene is expressed in all tissues during plant growth and development. There is no effective temporal or spatial regulation of target gene expression, which requires consumption of excessive matter and energy within the cells [33].

The CaMV35S promoter was isolated from a plant virus. As such, there are potential biosafety (in agriculture and human health) and environmental hazards involved. The risks that constitutive expression of viral capsid proteins in transgenic plants fall into three classes: transcapsidation, viral recombination to generate new strains of phytopathogens and synergism [34]. For instance, the CaMV35S promoter has a recombination hotspot [35] and is active in human enterocyte-like cells [36]; a plant virus could switch hosts to infect a vertebrate and then recombine with a vertebrate-infecting virus [37]. Overuse of the CaMV 35S promoter may lead to transcriptional inactivation [38,39]. The CaMV 35S also gives rise to the gene silencing phenomenon [40–45]. Because of these potential risks, research on novel plant sequences that function as promoter elements for moderate constitutive level expression of transgenes is becoming increasingly significant.

The CaMV 35S promoter is a strong promoter; not only does it affect the associated transgene, it can also affect genes thousands of base pairs up- or down-stream of the insertion site on a given chromosome or even affect the behavior of genes on other chromosomes. In *Brassica napus* plants containing the 35S promoter from the mosaic virus (CaMV), it has been shown that upon infection with CaMV the driven transgene is silenced [46]. CaMV is a common pathogen of *Brassica* sp., and oilseed rape genetically modified to be resistant to herbicide using the CaMV promoter loses that resistance when it encounters naturally CaMV, indicating that transgene phenotypes can be modified by pathogen invasion [40]. The presence of two or more chimeric genes in the same transformation vector driven by the same constitutive promoter in a single plant may result in homology-dependent gene silencing, particularly where the promoter is also highly active [47], and can involve interactions between closely linked repetitive elements on one chromosome or homologous sequences on separate chromosomes [42,45]. In this case, the reason for gene silencing is the inactivation of the CaMV 35S promoter [38,43]. In cases that introduce another gene into transgenic plants, if the resident transgene contains the CaMV 35S promoter, the introduction of additional copies of the 35S enhancer may result in methylation and silencing of unlinked homologous copies [41,44]. Thus, finding novel promoters for moderate constitutive level expression of transgene is becoming increasingly useful.

Generally, plant promoters are divided into three types: constitutive promoters, tissue-specific or development-stage-specific promoters and inducible promoters. The studies of plant constitutive promoters mainly focus on actin [19,20,25] and ubiquitin [7–11,24] gene promoters. The second type promoters direct the expression of gene in specific tissues or at certain development stages. For plants, promoters regulate the expression of genes in the photosynthetic tissues [48], vascular tissues [49], tubers [50,51], roots [50,52], endosperm [53], seeds [54] and reproductive organs [55,56]. Inducible promoters not conditioned to endogenous factors, but to environmental conditions, are promoters modulated by abiotic factors, such as drought [57], heat [58], cold [59,60] and wounding [61].

Poplar is a woody perennial that has been the subject of many studies focused on tissue-specific and inducible promoters [62–68], but relatively few studies about poplar constitutive promoters have been reported. There is a demand for moderate constitutive promoters; for instance, a very strong promoter would not be appropriate when expressing transgenes that are toxic or otherwise inhibitory in abundance [69]. Here, we cloned and analyzed a novel sequence from *P. tomentosa* that functions as a promoter element for moderate constitutive expression of transgenes, designated as the *PtMCP* promoter. A series of functional characterizations of the *PtMCP* promoter in stable *Arabidopsis* transformants were performed using RT-PCR, histochemical staining and fluorometric assays of GUS activity. We also report the expression patterns and quantitative measurements of GUS activity of the *PtMCP* promoter transiently expressed in tobacco.

## 2. Results and Discussion

### 2.1. Results

#### 2.1.1. Cloning and Sequence Analysis of the *PtMCP* Promoter

**Figure 1:**
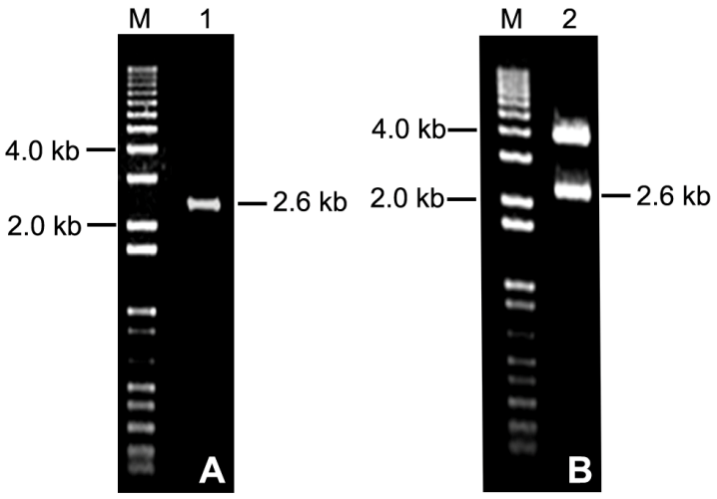
Cloning of the *PtMCP* promoter from *P. tomentosa*. (**A**) PCR product amplified from genomic DNA of poplar; (**B**) identification of the recombinant (*PtMCP* promoter-T construct) by enzyme digestion.

To examine the expression mechanism of the *PtMCP* promoter (GenBank accession number JX569147), a 2661 bp promoter fragment was isolated from genomic DNA of *P. tomentosa* (Figure 1). The putative transcription start site “A” was predicted by the TSSP-TCM software [70]. To look for putative *cis*-acting elements related to promoter function, the fragment was analyzed using the PLACE [71,72] and PlantCARE [73] databases. A TATA-box and a CAAT-box were located at positions 19 and 74 relative to the transcription start site. We also found several other putative *cis*-acting regulatory elements, including one TATC-box (28), one GARE-motif (473), one BoxI (587), one MBS (601), one TC-rich repeat (620), one Box4 (737), one GAG-motif (929), one TCA-element (1008), one CAT-box (1104), one Sp1 (1466), one TCT-motif (1527), one LTR (1613), two Box-W1s (1689, 1833), three ABRE/G-boxes (543, 576, 1628) and one CCGTCC-box/A-box (1334) (Figure S1).

#### 2.1.2. Construction and Transformation of the *PtMCP* Promoter::*GUS* Construct into *Arabidopsis*

**Figure 2:**
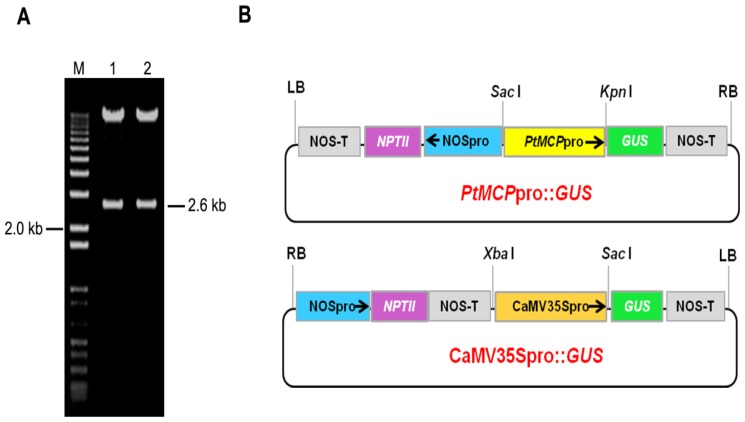
Construction of the *PtMCP* promoter::*GUS* construct. (**A**) Identification of recombinants (*PtMCP* promoter::*GUS* construct) by enzyme digestion; (**B**) Schematic diagrams of the *PtMCP* promoter::*GUS* and CaMV35S promoter::*GUS* construct. RB, right border; LB, left border; NOSpro, nopaline synthase promoter; *NPT-II*, neomycin phosphotransferase (II) coding region; NOS-T, nopaline synthase terminator; *GUS*, β-glucuronidase gene; *PtMCP*pro, *PtMCP* promoter; CaMV35Spro, cauliflower mosaic virus (CaMV) 35S promoter. The insertion position of the *PtMCP* promoter in the vector is indicated by the restriction enzyme sites *Sac*I and *Kpn*I.

To investigate the expression pattern of the *PtMCP* promoter, the isolated promoter fragment was fused with the *GUS* reporter gene in the pProtest vector to generate a *PtMCP* promoter::*GUS* construct (Figure 2). The resultant plasmid was stably introduced into *Arabidopsis* by the floral dip method. The binary vector pBI121, in which the *GUS* gene was driven by the cauliflower mosaic virus (CaMV) 35S promoter (Figure 2B), was transformed as the positive control, and wild-type *Arabidopsis* was used as a negative control. Consequently, we obtained 10 independent transgenic lines for *PtMCP* promoter::*GUS*. Of these, eight independent transgenic lines were used for further analysis.

#### 2.1.3. Characterization and Analysis of Transgenic *Arabidopsis*

**Figure 3:**
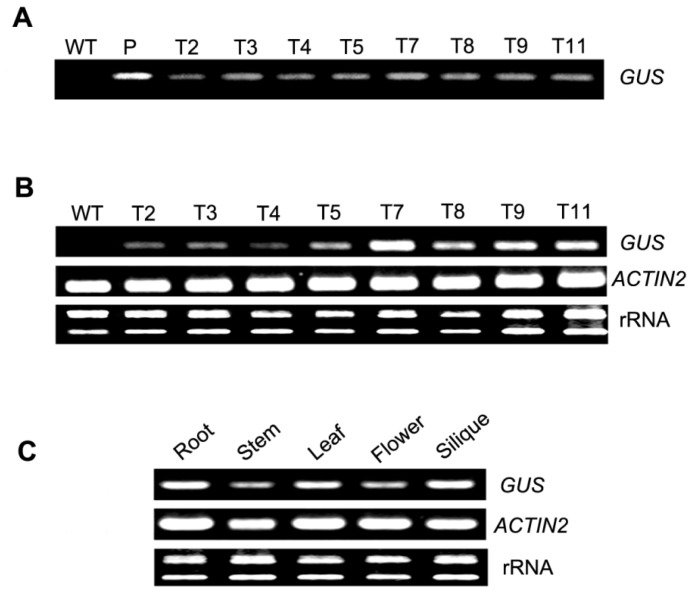
Characterization and analysis of transgenic *Arabidopsis*. Analysis of the different transgenic *Arabidopsis* lines (T2–T5, T7–T9, T11) by targeting the *GUS* reporter gene in (**A**) genomic PCR, (**B**) RT-PCR and (**C**) *GUS* transcript abundance in different tissues (root, stem, leaf, flower, silique) of transgenic *Arabidopsis* plants by RT-PCR. WT, wild-type plants serving as negative controls; P, positive control (*PtMCP* promoter::*GUS* plasmid). The *Arabidopsis ACTIN2* gene was used as an endogenous reference gene for the RT-PCR assay and also amplified to verify that similar amounts of cDNA were used. The RT-PCR data are representative of at least three experiments. Numbers on the left represent the size of the amplified cDNA fragments in base pairs.

The stable integration of T-DNA into the genome of the transgenic plants was verified by genomic PCR analysis using *GUS* gene-specific primers. An expected 239 bp specific amplification product for the *GUS* gene was obtained from all eight transgenic lines tested (T2–T5, T7–T9, T11), and no amplification signal was observed in the wild-type control plants (Figure 3A). Furthermore, RT-PCR using *in vitro* grown plants demonstrated that the transcript of the *GUS* reporter gene was present in the eight transgenic lines; the *GUS* transcript was not detected in wild-type control plants. Notably, the GUS expression levels varied among different transgenic lines, and RT-PCR revealed that the transcript abundance of *GUS* in all transgenic lines was lower than that of the reference *ACTIN2* gene. The *GUS* transcript abundance of the T7 transgenic line was the highest (Figure 3B). To determine the tissue-specificity of the *PtMCP* promoter in *Arabidopsis*, RT-PCR was performed. RT-PCR indicated that the *GUS* reporter gene was transcribed in the root, stem, leaf, flower and silique of *Arabidopsis*. The *GUS* transcript abundance was similar among the various tissues and organs and was lower than that of the reference *ACTIN2* gene (Figure 3C).

#### 2.1.4. Histochemical and Fluorometric GUS Assays

**Figure 4:**
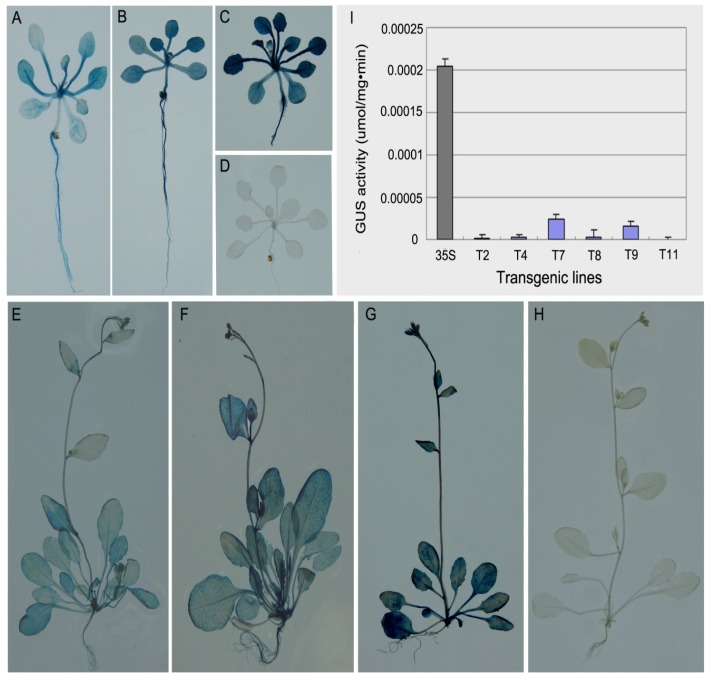
Histochemical and fluorometric GUS activity assays. Histochemical GUS staining of transgenic lines (T2 and T7) containing the *PtMCP* promoter::*GUS* construct in *Arabidopsis* seedlings (**A**–**D**) and maturing plants (**E**–**H**). GUS activity was visible in all tissues, including roots, stems, rosette leaves, cauline leaves and flowers, but was weaker than in CaMV35S promoter::*GUS* plants. (**A** and **E**), transgenic line 2 (T2); (**B** and **F**), transgenic line 7 (T7); (**C** and **G**), positive controls (CaMV35S promoter::*GUS* plants); (**D** and **H**), negative controls (wild-type plants); (**I**) Quantitative measurement of GUS activity in different transgenic lines (T2, T4, T7, T8, T9 and T11) and positive control (CaMV35S promoter::*GUS* plants). Error bars represent the standard deviation (SD). Data are the mean values of triplicate tests ± SD (*n* = 3).

To understand the spatial expression pattern of the *PtMCP* promoter, histochemical GUS activity was analyzed in different tissues and organs of transgenic *Arabidopsis* seedlings and maturing plants (T2, T7) in the absence of environmental influences. Histochemical staining showed moderate GUS activity in the roots, stems and leaves of seedlings and the roots, stems, rosette leaves, cauline leaves and flowers of maturing plants, but it was weaker than in the corresponding tissues of positive controls (CaMV35S promoter::*GUS*). There was no GUS activity observed in these tissues in the negative controls (wild-type plants) (Figure 4A–H). Quantitative measurement of GUS activity also showed that GUS activity in the *PtMCP* promoter::*GUS* plants (T2, T4, T7, T8, T9 and T11) was significantly lower than in the CaMV35S promoter::*GUS* plants (Figure 4I). These results indicated that the *PtMCP* promoter was constitutive, but its expression intensity was significantly lower than the CaMV35S promoter.

#### 2.1.5. Transient Expression of the *PtMCP* Promoter in Tobacco

**Figure 5:**
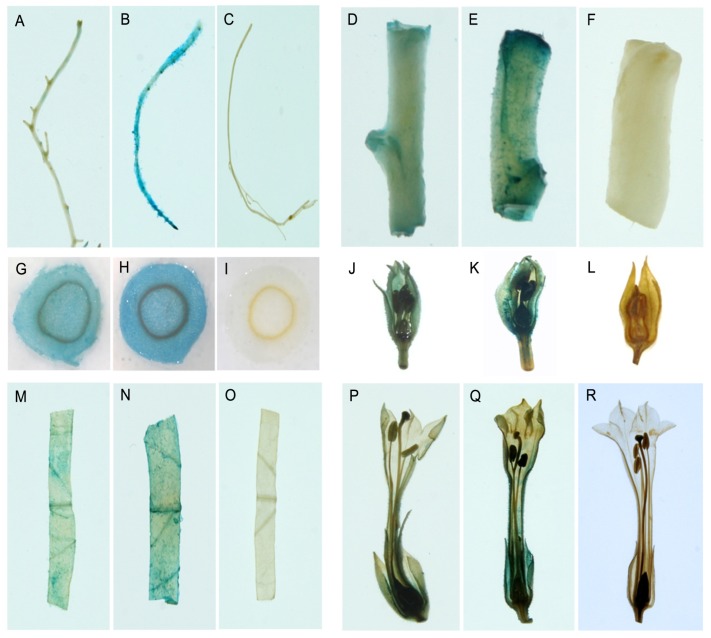
Histochemical GUS staining of different tissues of tobacco transiently transformed with an *Agrobacterium* suspension. First and fourth panels (**A**, **G**, **M**, **D**, **J**, **P**), *PtMCP* promoter::*GUS* tobacco; second and fifth panels (**B**, **H**, **N**, **E**, **K**, **Q**), positive controls (CaMV35S promoter::*GUS* tobacco); third and sixth panels (**C**, **I**, **O**, **F**, **L**, **R**), negative controls (wild-type tobacco). (**A**–**C**) roots; (**D**–**F**) stems; (**G**–**I**) cross-section of stems; (**J**–**L**) flower buds; (**M**–**N**) leaves; (**P**–**R**) flowers. Regions positive for GUS appear blue.

**Figure 6:**
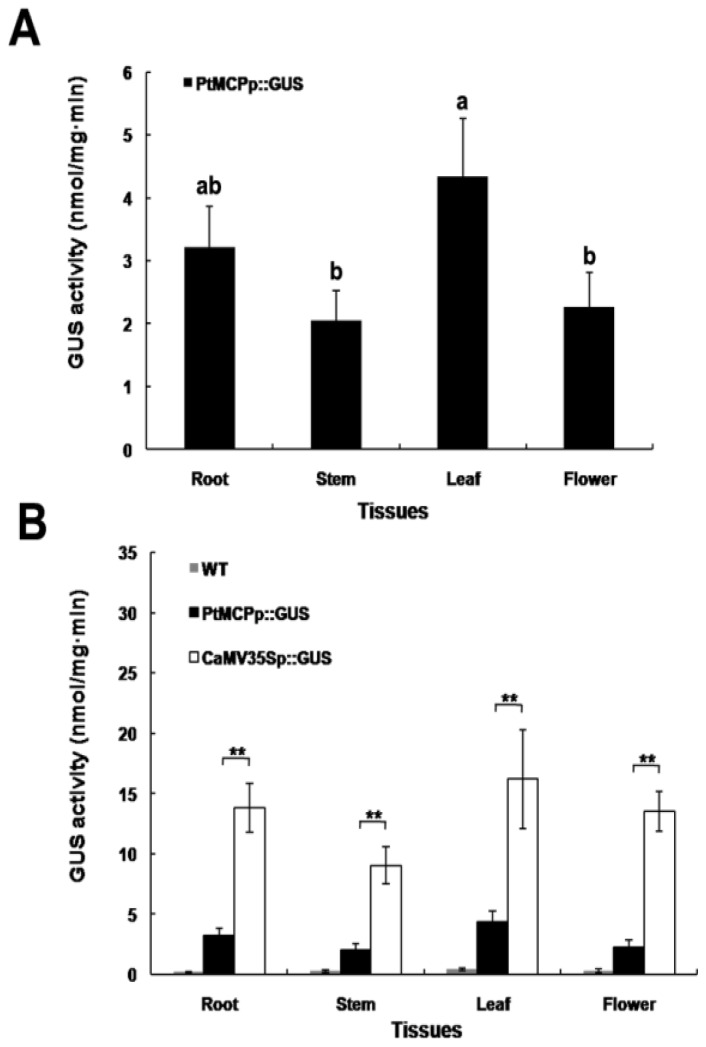
Quantitative measurement of GUS activity in different tissues of *PtMCP* promoter::*GUS* tobacco and positive controls (CaMV35S promoter::*GUS* plants). GUS activity was determined using protein extracts from different tissues. (**A**) Quantitative analyses of GUS activity in different tissues and organs of *PtMCP* promoter::*GUS* plants. Letters above the columns indicate statistically significant differences (*p < 0.05) according to the ANOVA Fisher’s LSD test. Error bars represent the standard deviation (SD). Data are the mean values of triplicate tests ± SD (*n* = 3); (**B**) Quantitative measurement of GUS activity in different tissues of *PtMCP* promoter::*GUS* and CaMV35S promoter::*GUS* tobacco. Significance was assessed using Student’s *t*-tests (**p < 0.01).

To determine the spatial expression of the *GUS* gene under the control of the *PtMCP* promoter in tobacco, GUS activity following *Agrobacterium*-mediated transient expression was analyzed in different tissues in the absence of environmental influences. Histochemical staining showed that the *PtMCP* promoter directed moderate expression of the *GUS* reporter gene in all tobacco tissues examined, including the root (Figure 5A), stem (Figure 5D,G), leaf (Figure 5M), flower bud (Figure 5J) and flower (Figure 5P), but it was weaker than the GUS activity in the corresponding tissues and organs of positive controls (CaMV35S promoter::*GUS*) (Figure 5B,E,H,K,N,Q). No visible GUS activity was detected in these tissues and organs in wild-type plants (Figure 5C,F,I,L,O,R). To increase the sensitivity of *GUS* reporter gene detection, quantitative measurements were made (Figure 6). Moderate GUS activity was detected in the root, stem, leaf and flower, but the protein levels differed in the various tissues. The maximum amount of GUS protein was detected in the leaf, while levels were lowest in the stem (Figure 6A). GUS activity in the *PtMCP* promoter::*GUS* plants was significantly lower (*p* < 0.01) than in corresponding tissues of CaMV35S promoter::*GUS* plants. GUS activity in the negative controls (wild-type plants) was negligible (Figure 6B). These results were identical with the results in *Arabidopsis* (Figure 4); the *PtMCP* promoter was moderately constitutive. Moreover, a similar expression pattern was also observed in poplar (data not shown).

### 2.2. Discussion

To investigate the expression mechanism of the *PtMCP* promoter, a 2661 bp sequence was isolated and analyzed in this study. The spatial and temporal expression patterns of this promoter were first clarified by assaying GUS activity in transgenic *Arabidopsis* plants containing a *PtMCP* promoter::*GUS* fusion construct. The construct was stably transformed into *Arabidopsis* and transiently introduced into tobacco to examine whether the function of this promoter is conserved between different species.

Using the PLACE [71,72] and PlantCARE [73] databases, putative TATA and CAAT-boxes were located at positions 19 and 74 relative to the transcription start site “A”, and several other common *cis*-elements were also identified in the *PtMCP* promoter. The G-box motif is a light responsive *cis*-acting element that has been found to be essential for transcriptional activity in the *Arabidopsis rbcS-1A* promoter [74], the spinach ribulose bisphosphate carboxylase/oxygenase (RuBisCO) activase promoter [75] and the rice *OsActin2* gene regulatory region [25]. TC-rich repeats are involved in defense and stress responses and are also found in yeast [76] and barley [77] ubiquitin extension protein promoters. The TATC-box and the GARE-motif are involved in gibberellin-responsiveness, BoxI, Box4, the GAG-motif, Sp1 and the TCT-motif are light responsive elements, MBS is an MYB binding site involved in drought-inducibility, TCA-elements are involved in salicylic acid responsiveness, the CAT-box is related to meristem expression, LTR is involved in low-temperature responsiveness, Box-W1 is a fungal elicitor responsive element and ABRE is involved in abscisic acid responsiveness.

In transgenic *Arabidopsis*, histochemical GUS staining showed GUS activity in all tissues and organs (roots, stems, rosette leaves, cauline leaves and flowers) of seedlings and maturing plants, but it was weaker than the GUS activity in CaMV35S promoter::*GUS* plants. Furthermore, quantitative measurement of GUS activity also proved that GUS activity in the *PtMCP* promoter::*GUS* plants was significantly lower than in CaMV35S promoter::*GUS* plants. These results were in agreement with the RT-PCR results; RT-PCR indicated that the *GUS* reporter gene was transcribed in the root, stem, leaf, flower and silique of *Arabidopsis*. The reason why different *GUS* expression levels among transgenic *Arabidopsis* lines may be the T-DNA insertion position in plant genome and different copy number.

To contrast with the expression pattern in *Arabidopsis*, the same *PtMCP* promoter::*GUS* construct was transformed into tobacco transiently, and a similar expression pattern was observed. Histochemical GUS staining showed GUS activity in all tissues and organs of tobacco, including the root, stem, leaf, flower bud and flower, but it was weaker than the GUS activity in CaMV35S promoter::*GUS* plants. Quantitative analyses showed that GUS activity in the *PtMCP* promoter::*GUS* plants was significantly lower (*p* < 0.01) than in the corresponding tissues and organs of CaMV35S promoter::*GUS* plants.

## 3. Experimental Section

### 3.1. Plant Materials and Growth Conditions

The poplar (*Populus tomentosa* Carrière) clone TC1521 was used as source material for this study. Poplar plants were grown in a growth chamber under long-day conditions (16-h light/8-h dark, cool white fluorescent light, minimum illumination: 0.2 mM/s·m^2^) at 22 °C. Four-month-old plants with an identical growth status were then used for young leaf collection and prepared for cloning of the *PtMCP* promoter.

Plants of *Arabidopsis thaliana* L. ecotype Columbia (Col) used in plant transformation were grown and maintained in the greenhouse of the National Engineering Laboratory for Tree Breeding, Beijing Forestry University, Beijing. *Arabidopsis* seeds were sterilized and placed on 1/2 Murashige-Skoog (MS) solid medium [78] at 4 °C for 2–4 days. The seedlings were grown in a growth chamber under the same conditions as poplar plants for 10 day before being transplanted to artificial soil mix composed of humus, vermiculite and perlite [1:2:1 (*v*/*v*/*v*)].

Tissue-cultured tobacco plants (*Nicotiana tabacum* cv. W38) were raised and synchronized (using vegetative stem cuttings containing an axillary bud) on 1/2 Murashige-Skoog (MS) solid medium [78] supplemented with 0.4 mg/L IBA and adjusted to pH 5.8. *In vitro* and tissue-cultured tobacco plants were grown in a growth chamber under the same conditions as poplar plants before being used for *Agrobacterium*-mediated transient expression assays.

### 3.2. Bacterial Strains

*Escherichia coli* (strain DH5α) was used for the cloning and propagation of all recombinant plasmid vectors. *Agrobacterium tumefaciens* (strain GV3101) was used to transform *A. thaliana* (Col) plants via the floral dip method [79,80]. The plasmids pProtest (Obtained from the Laboratory of Professor Steven H. Strauss, Oregon State University, Corvallis, OR, USA), pMD19-T vector and pBI121 (Clontech, Mountain View, CA, USA) were used to generate promoter fragment constructs.

### 3.3. Construction of the PtMCP Promoter::GUS Plasmid

To construct a binary vector consisting of the GUS coding sequence driven by the *PtMCP* promoter, a fragment of approximately 2.6 kb was obtained by PCR amplification using *P. tomentosa* genomic DNA as a template. Genomic DNA was extracted from leaves (300 mg) using a modified CTAB extraction method, as previously described [81]. Primers with additional restriction sites were designed: The forward primer 5′-CTACGAGCTCTACTAAATAAATATATAA-3′ and the reverse primer 5′-ATGGTACCATCTATCTGCCCCCTTGTC-3′. The restriction enzyme sites *Sac*I (in the forward primer) and *Kpn*I (in the reverse primer) are underlined. PCR reactions were carried out in 20 μL volumes under the following conditions: 94 °C for 3 min; 35 cycles of 94 °C for 30 s, 57 °C for 30 s and 68 °C for 2.5 min. PCR products were subcloned into the pMD19-T vector (TaKaRa, Otsu, Japan) and then sequenced. The promoter fragment was analyzed using the PLACE [71,72] and PlantCARE [73] databases to find putative functional promoter elements. The *PtMCP* promoter-T construct was digested with *Sac*I/*Kpn*I and then cloned into the corresponding site of the expression vector pProtest. The resultant *PtMCP* promoter::*GUS* construct was transformed into *A. tumefaciens* strain GV3101 by electroporation [82].

### 3.4. *Arabidopsis* Transformation

*A. tumefaciens* strain GV 3101 containing the *PtMCP* promoter::*GUS* construct was used to transform *A. thaliana* (Col) plants via the floral dip method [79,80]. Kanamycin (50 μg/mL) resistant transformants (T0) were transplanted from the proliferating medium to artificial soil mix and grown in a growth chamber set at 22 °C under LD conditions. Further analyses were performed, too.

### 3.5. Genomic PCR and RT-PCR Assays

For preliminary verification of the presence of transgenes, putative transgenic plants were screened by PCR. Total genomic DNA was extracted from all of the kanamycin-resistant plants with a Plant Genomic DNA Kit (TIANGEN, Beijing, China). PCR was performed in a total volume of 20 μL, with 2 μL of 10× PCR buffer, 1.6 μL of 2.5 mM dNTPs, 2 μL plant genomic DNA(100 ng/L) 0.4 μL each of forward and reverse primers (10 μM), 0.2 μL Taq DNA polymerase (5000 U/mL) and 13.4 μL ddH_2_O. Thermal cycling was performed at 94 °C for 5 min, then 94 °C for 30 s, 61 °C for 15 s and 72 °C for 20 s, for 30 cycles, with final extension at 72 °C for 5 min and, finally, kept at 4 °C, using the GeneAmp^®^ PCR System 9700 (ABI, Hong Kong, China). Genomic PCR was carried out using *GUS* gene-specific primers: forward primer 5′-GTTACGTCCTGTAGAAACCCCAACC-3′ and reverse primer 5′-CTGCCCAACCTTTC GGTATAAAGAC-3′.

Total RNA was extracted from *Arabidopsis* samples, according to the method described previously [83]. Total RNA was pre-treated with RQ1 DNase I (Promega, Madison, WI, USA) to eliminate residual genomic DNA. First-strand cDNA was synthesized using 1.0 μg of DNase-treated total RNA, Superscript III (Invitrogen, Carlsbad, CA, USA) and oligo d(T)_20_ in a total volume of 20 μL. The first-strand cDNA was diluted 1:10 with ddH_2_O, and 2 μL of the diluted cDNA was used as a template for RT-PCR analysis. RT-PCR was performed in a total volume of 20 μL. *GUS* primers: forward primer 5′-GTTACGTCCTGTAGAAACCCCAACC-3′ and reverse primer 5′-CTGCCCAACC TTTCGGTATAAAGAC-3′. The *A. thaliana ACTIN2* gene (Phytozome v8.0 accession: AT3G18780) was used as an endogenous control gene with the primers: forward 5′-AAGCACA ATCCAAGAGAGGTATTC-3′ and reverse 5′-TACATAGCGGGAGAGTTAAAGGTC-3′. To detect expression patterns and transcript levels of the *PtMCP* promoter in *Arabidopsis*, total RNA was extracted from various tissues (root, stem, leaf, flower, silique). The expression pattern of *PtMCP* promoter in *Arabidopsis* was then analyzed by RT-PCR. PCR products were evaluated following agarose gel electrophoresis and spectrophotometrical analysis.

### 3.6. Histochemical and Fluorometric GUS Assays

The Histochemical staining of GUS activity was performed according to the method described previously [84]. Transgenic *Arabidopsis* samples were immersed into GUS reaction buffer [1 mM X-Gluc (5-bromo-4-chloro-3-indolyl-β-d-glucuronide), 100 mM phosphate buffer pH 7.0, 0.1% Triton X-100, 5 mM K_3_Fe(CN)_6_, 5 mM K_4_Fe(CN)_6_, 10 mM EDTA and 20% methanol]. After overnight incubation in dark at 37 °C, stained samples were bleached with 70% (*v*/*v*) ethanol and then observed.

Fluorometric assay for GUS activity was performed according to the method of Jefferson [84]. The transgenic plants were ground in liquid nitrogen and homogenized in freshly prepared GUS extraction buffer (50 mM phosphate buffer, pH 7.0, 10 mM EDTA, 0.1% Triton X-100, 0.1% (*w*/*v*) sodium lauryl sarcosine, 10 mM β-mercaptoethanol). The homogenate was then centrifuged for 10 min at 12,000 rpm at 4 °C, and the GUS activity of the supernatant was assessed using 4-methylumbelliferyl glucuronide (4-MUG) as a substrate. The fluorescence of the GUS-catalyzed hydrolysis reaction product, 4-methylumbelliferone (4-MU), was measured with the TECAN GENios system (Tecan, Shanghai, China). The protein concentration in the supernatant was determined by the procedure of Bradford [85], using bovine serum albumin (BSA) as a standard. GUS activity was normalized to the protein concentration of each supernatant extract and expressed as picomoles 4-MU per minute per milligram protein.

### 3.7. *Agrobacterium*-Mediated Transient Expression in Tobacco

For *Agrobacterium*-mediated transient expression of the *PtMCP* promoter in tobacco, root, stem, leaf, flower bud and flower tissues were infiltrated into *Agrobacterium* culture (OD_600_ = 0.8). After 20–30 min of vacuum filtration, the infiltrated samples were blotted dry with sterile filter paper and maintained in petri-dishes containing co-cultivation medium (MS medium) at 25 °C in a dark place for 48 h. The samples were then collected and subjected to histochemical and fluorometric GUS assays.

## 4. Conclusions

A novel constitutive promoter with moderate activity from *P. tomentosa*, designated the *PtMCP* promoter, has one TATA-box, one CAAT-box and several other putative *cis*-acting regulatory elements. The expression mechanism of the *PtMCP* promoter is similar to that of the CaMV 35S promoter, but the levels of activity are considerably lower. The function of the *PtMCP* promoter from poplar is presumably conserved in different species. Therefore, the *PtMCP* promoter described in this paper provides a practical choice to direct moderate level constitutive expression of transgenes. The emergence of this promoter could perhaps provide a valuable new tool for scientists in plant genetic engineering.

## Supplementary Information
