# Supplementary material for: A Novel Moderate Constitutive Promoter Derived from Poplar (Populus tomentosa Carrière)
Source: Int J Mol Sci. 2013 Mar 18;14(3):6187–204. doi: 10.3390/ijms14036187 (PMC3634493; doi:10.3390/ijms14036187)
Supplement: Supplementary file 1 [file ijms-14-06187-s001.docx]

Supplementary Information

**Figure S1.** Nucleotide sequence of the *PtMCP* promoter. The putative transcription start site (TSS) is designated +1 and denoted by an asterisk. The TATA and CAAT-boxes are boxed; all putative *cis*-acting elements are indicated and underlined.


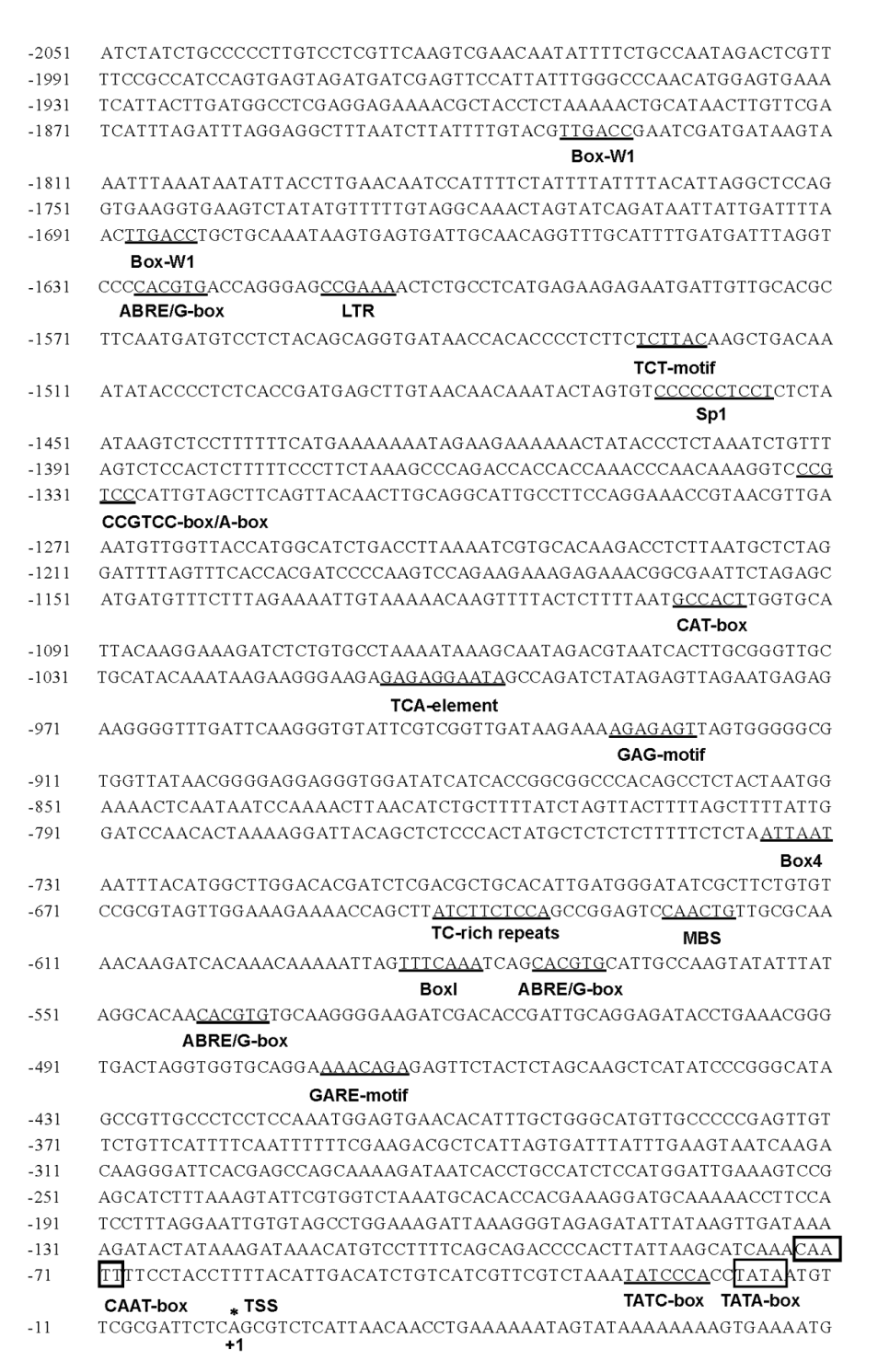


© 2013 by the authors; licensee MDPI, Basel, Switzerland. This article is an open access article distributed under the terms and conditions of the Creative Commons Attribution license (http://creativecommons.org/licenses/by/3.0/).
